# An analysis of changes in mobility and safety of older drivers associated with a specific older driver on-road licensing test: a population study

**DOI:** 10.1186/1471-2458-14-165

**Published:** 2014-02-14

**Authors:** Michael D Keall, Esther Woodbury

**Affiliations:** 1 Department of Public Health, University of Otago, Wellington, New Zealand

**Keywords:** Older driver safety, Licensing policy, On-road driving tests, Mobility

## Abstract

**Background:**

From 1999 to the end of 2006, older drivers aged 80 plus in New Zealand were subject to an older driver licence test as a condition of licensing. The impact of this test has not yet been studied in terms of the safety and mobility of older people.

**Methods:**

Three main data sources were analysed to provide evidence of changes in older driver travel mode choice, licensing rates and injuries: New Zealand Travel Survey data, licensing data and police-reported crash data.

**Results:**

General trends towards higher levels of motorised mobility were apparent for this group over the 20 years studied, but without an obvious change at the points in time when the test was either introduced or removed as a general requirement of licensure. There were also no apparent changes in the levels of pedestrian activity or road injury involving drivers in this age group.

**Conclusions:**

Along with other relevant studies comparing older driver licensing policies across different jurisdictions, this study does not support the generalised use of on-road testing as an assessment mechanism for all older drivers.

## Background

Older drivers have elevated crash rates in many countries, which can be used as a justification of specific licensing conditions targeted to this group, including on-road driving tests. In New Zealand, an older driver on-road driving test was introduced in 1999, which, together with certification of medical fitness, was a biennial requisite of driver licensing for drivers aged 80 and over. Following a medical check that health and eyesight were satisfactory for driving, the older driver undertook an on-road test of about 20 minutes’ duration in which their ability to detect traffic hazards around the vehicle, as well as their ability to control the vehicle and adhere to the road rules, was assessed. The driving test was failed if the driver committed major errors (e.g., failure to stop or maintain vehicle position in the lane) or a combination of more minor errors.

This test has been shown to provide some predictive value for crash involvement: a study showed that failures in the driving test were associated with higher crash involvement rates, controlling for relevant other factors such as driver age [1]. Nevertheless, the generalised use of the test was dispensed with in December 2006, a decision supported by local and international evidence that, per licensed driver, older drivers on average do not have elevated crash involvement rates, particularly when taking into account their propensity to be injured when crash-involved [2-5].

From 2007 onwards, drivers at age 75, 80 and every two years after that were still required to obtain a medical certificate from their general practitioner (GP), who could make the following recommendations: the patient is medically fit to drive without conditions imposed; the patient is medically fit to drive with specified conditions (such as no night driving; only driving within 10 km of their home); the patient is medically fit to drive but must undergo an on-road driving test; the patient requires further specialist assessment (a medical specialist or an occupational therapist) before they can be deemed medically fit to drive; the patient is not medically fit to drive. Previous analysis of older driver failure rates found that about 5% of drivers aged 80 plus who sought to be licensed failed the on-road driving test, often after more than one attempt [1]. Under the new licensing system (from 2007 onwards), these drivers would presumably still be driving unless identified by their GP as being unsafe.

When it was compulsory, the on-road driving test was reportedly experienced as very stressful for older drivers, and was often cited as a reason for relinquishing the driving licence, leading to an impaired level of independent mobility for many [6]. Driving cessation is strongly associated with decreased out-of-home activity after adjusting for sociodemographic and health-related factors [7]. Depressive symptoms can arise in response to consequent reduced access to resources such as paid or voluntary work, health care services, social contacts, etc. [8]. A six-year US study found that driving cessation was the strongest predictor of depression among a sample of older people after adjusting for sociodemographic and health-related factors [9]. Relatedly, Metz [10] found that a lower level of mobility was associated with a decrease in quality of life, particularly when drivers were already affected by age-associated disability. Marottoli et al. [9] hypothesised that for people with mobility issues, a sense of control over their environment is severely impaired by the lack of ability or opportunity to drive.

Removing the test requirement for older people might cause a fall in active travel (particularly walking) as a proportion of mode share, but time spent walking could feasibly increase due to higher levels of out-of-home activity [7]. For example, upon driving cessation, older people who previously drove to undertake shopping may no longer do their own shopping, thus relinquishing walking activity that would have taken place to and from their car and around the grocery store. Nevertheless, an analysis of New Zealand Travel Survey data for people aged 75 plus who had ceased to have access to a car found that walking and passenger trips grew on average as a proportion of mode share, but mobility overall decreased [11]. It would therefore be expected that the removal of the compulsory on-road driving test would have two main consequences for older people: improved access to motor vehicles; and consequent higher crash rates. The higher crash rates would arise from an increase in the amount of driving undertaken by this group, but could potentially be elevated further by allowing higher risk drivers to remain licensed (who would otherwise have failed the on-road licensing test). Some previous studies have compared older driver safety in jurisdictions with different older driver licensing policy [12,13], finding little safety benefit from stricter regulation. However, no studies to our knowledge have looked at changes occurring in the same jurisdiction before and after a significant policy change. The current study aimed to identify any changes in licensing rates, crash rates, amount of driving and pedestrian activity by the group affected (New Zealand residents aged 80 plus) in comparison with other age groups, associated with the on-road driving test licensing requirement over the period 1999 to 2006.

## Methods

Three main data sources were analysed to provide evidence of changes in older driver travel mode choice, licensing rates and injuries: New Zealand Travel Survey data, licensing data and police-reported crash data.

### New Zealand travel survey

The New Zealand Travel Survey has been run for single years, from mid-1989 to mid-1990, similarly in 1997/98, and then has been run continuously since 2003 [14,15]. Using in-person interviews, supported by travel diaries, the survey collects detailed travel behaviour data for two specified days of the year from all occupants of randomly sampled households. Annual travel is then derived from the particular travel days by ensuring that the travel days are spread throughout the year and there is minimal geographic clustering of surveying that could lead to biases. Travel activity as drivers, passengers, cyclists and pedestrians can be estimated from these data. Analysis carried out in this study used published estimates from the travel surveys [14-17]. Sample sizes of older people in these surveys were relatively small. For example, in the 1997/98 survey, there were 657 respondents who were aged 75 plus (5% of the sample), of which 305 were aged 80 plus (2% of the sample) [14]. Sampling errors have not been published for all the surveys, but they will be relatively large for small samples. For example, for sample sizes of around 600, the 1997/98 survey total distance driven was estimated to be between plus and minus 10%-13% of the estimated total with 95% confidence [14].

### Licensing data

A national register of licensed drivers is held by the New Zealand Transport Agency (NZTA), allowing analyses of the numbers of licensed drivers according to age, year of licensing, and other variables. Data was requested from the NZTA on the numbers of drivers passing the older driver on-road driving test. Information on the total numbers of licensed drivers is available in the annual statistical statements published by the Ministry of Transport (e.g. [18]).

### Crash data

The Ministry of Transport maintains the Crash Analysis System [19], which records details of police-reported crashes involving motor vehicles in which a medically treated injury occurred. Such crashes are legally required to be reported to police. Although crashes involving a death can be assumed to be completely reported, there is underreporting of crashes resulting in the hospitalisation of a road user. Alsop and Langley [20] estimated that less than two-thirds of all hospitalised vehicle occupant traffic crash victims were recorded by the police. They also found that reporting rates varied significantly by age (but mainly for children rather than older adults), injury severity, month of crash, number of vehicles involved (with better coverage of multi-vehicle collisions), and geographic region. They found no systematic reporting differences by gender or ethnicity of the victims, or day of the week. Minor injuries (those requiring medical attention, but not admission to hospital) are likely to show similar reporting patterns, but have still poorer coverage, as lower degrees of injury severity were accompanied by lower rates of reporting. Road crash and injury data analysed for this study were obtained with permission from the Ministry of Transport.

## Results

The Results section analyses changes in licensing rates, crash rates, amount of driving and pedestrian activity by the group affected (New Zealand people aged 80 plus) by the on-road testing component of licensing policy in comparison with other unaffected age groups.

**Figure 1:**
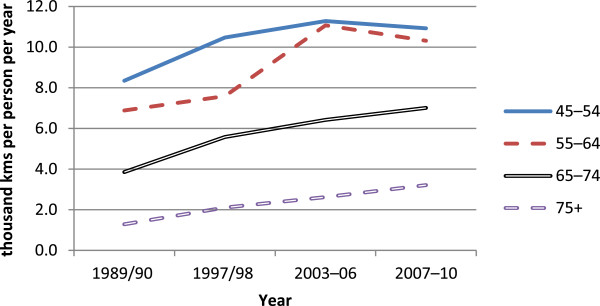
**Annual distance driven per capita (thousand kms) in light 4-wheeled vehicles, by year and age group.** Source: NZ Travel Survey [17]; Statistics NZ [21].

**Figure 2:**
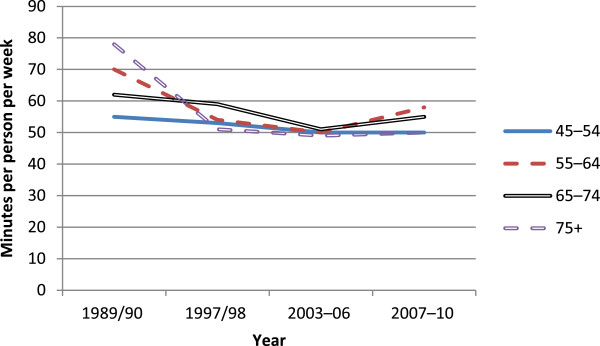
Estimated number of minutes spent walking per person per week by age group.

Figure 1 shows estimates of distance driven per person in light 4-wheeled vehicles published by the New Zealand Ministry of Transport [17], by year and particular age groups to show how driving activity may have changed around the time of the licensing regulation change from 1999 to the end of 2006. The age group 75 and over includes a large proportion of drivers unaffected by the regulation change. For example, in 2004, licensed drivers aged 75–79 constituted 60% of all licensed drivers aged 75 plus [18]. The estimates are of estimated distance driven as derived from Travel Survey data [17], divided by population estimates [21]. A striking feature of the figure is the relatively small amount of driving undertaken by those aged 75 plus. It is also apparent that this group’s per person driving activity increased substantially over the period shown, almost trebling from 1989/90 (prior to the introduction of the on-road licensing test) to 2007/10 (after the test as a general requirement was removed). Over the period immediately before and immediately after the on-road test licensing requirement was removed (2003/06 and 2007/10, respectively) per person driving distances for people aged 75 plus increased from around 2,600 km to around 3,200 km, an increase of about 20%.Figure 2 shows the estimated average number of minutes spent walking per week per person as derived from the New Zealand Travel Surveys over the period 1989 to 2010. An examination of the change in walking before, during (1999 to the end of 2006) and after the regulation change for the age group 75+, which includes the age group 80 plus affected by the regulation, shows little change in walking for this age group even though the age groups 55–64 and 65–74 show an apparent decrease prior to the regulation followed by an increase subsequently, which may indicate some background changes in propensity to walk. Sampling errors for these estimates were not published, so confidence intervals cannot be placed around the point estimates.

Table 1 shows numbers of licensed drivers aged 80 plus as at July of the year specified, along with the proportion of drivers who were licensed via the on-road test. Note that as the licence for older drivers is renewed every two years, the 2007 figures include in the last column all drivers who sat the on-road test in the previous year. It is clear that the on-road test played a relatively insignificant role in the licensing of drivers aged 80 plus from 2008 onwards, although the percentages shown are based on numbers of on-road licensing tests undergone by all drivers aged 75 plus, and are therefore somewhat inflated.

**Table 1:** Number of licensed drivers aged 80 plus, with percentage that underwent an on-road test as a condition of their licensing, as at July of year specified

**Year**	**Total licensed**	**Proportion who sat on-road test**
2005	54335	100%
2006	57339	100%
2007	62431	46%*
2008	67756	1%*
2009	71383	1%*
2010	77953	1%*

*the actual percentages will be somewhat lower than stated as the statistics provided on numbers sitting the test included drivers aged 75–79.

**Figure 3:**
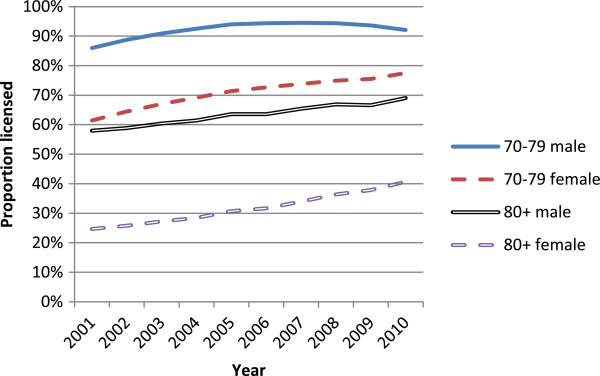
Proportion of given and sex group that were licensed by year.

**Figure 4:**
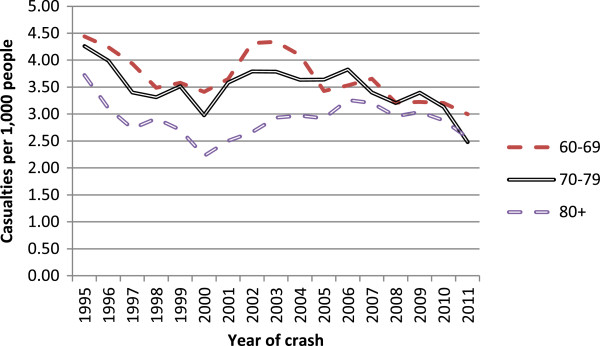
Number of casualties occurring in crashes per year in which given age group was driver.

**Figure 5:**
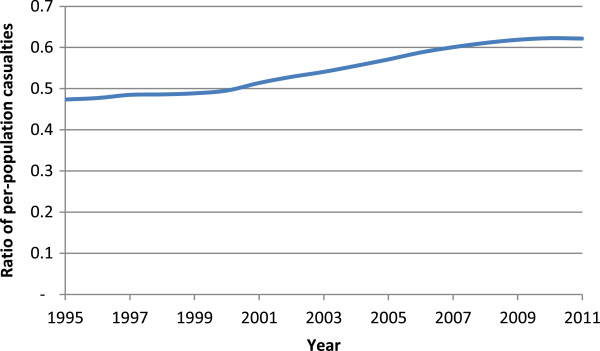
Proportion of casualties occurring in crashes involving drivers aged 70 plus that involved drivers aged 80 plus, by year.

Table 1 shows general increases in licensed older drivers per year. As the population ages, demographic changes would lead to increases in numbers licensed, so it is informative to look at the proportion of the population that is licensed to drive. These are shown in Figure 3. The administrative data allow us to look at licensing rates for males and females separately, an important disaggregation of the data as older females have traditionally had very low licensing rates, although they were increasing more rapidly over the period shown relative to their male counterparts. The increases in the licensing rate per population over the period shown were 65% for females aged 80 plus, 26% for females aged 70–79, 19% for males aged 80 plus, and 7% for males aged 70–79.Figure 4 shows the number of casualties occurring in reported injury crashes by the age group of the driver per thousand population in that age group. Note that in a two-car crash, for example all casualties are counted twice, once per driver. This figure gives an indication of the injury burden associated with different driver age groups, without considering attribution of fault. Note that the period studied includes an economic crisis whose effects started to be felt from 2008 onwards, which reduced levels of mobility generally in New Zealand, as in many countries. Looking at the series for drivers aged 80 plus, there appears to be no change towards higher levels of safety over the period 1999 to 2006 when the on-road licensing test was a licensing requirement.A potential comparison group for drivers aged 80 plus is the age group 70–79, whose mobility (and resulting crash rate) is likely to be affected in a similar way by changes in economic factors or policies over time. Figure 5 uses this group as a comparison group to highlight potential changes in casualty rates that might be associated with the on-road licensing test for drivers aged 80 plus from 1999 the end of 2006. The ratio of casualties per population for the age group 80 plus as drivers divided by the same measure for the age group 70–79 do not show any reductions over this period that might correspond to improved safety levels or reduced mobility at a population level. There was a generally upward trend with time, steepening somewhat from around 2001 (two years after the on-road test became a requirement for the licensing of drivers aged 80 plus), which then levels out after 2008. The increasing trend reflects increasing licensing rates for drivers in the older age group, as shown in Figure 3, above.

## Discussion

To examine the potential public health impact of a change in licensing policy for older drivers, licensing data, travel activity estimates and crash data were all analysed, looking for step changes at the time of the policy change. The analysis described above did not identify any such changes. The proportion of the age group affected who were licensed to drive continued to grow. The amount of driving undertaken by people aged 75 plus increased substantially over the two decades studied, approximately quadrupling from 1989/90 to 2007/10, but without an apparent change in activity over the period of the licensing policy change. There was a general decrease in the amount of time spent walking by this age group, but little evidence that there was any real decrease in walking activity that could be hypothesised to accompany a significant mode shift to or from motorised travel at the times of policy change. The numbers of casualties arising from crashes involving drivers aged 80 plus changed over time in a way that was consistent with changes seen for the adjacent age group not affected by the policy, drivers aged 70–79, again providing no evidence of a safety change associated with the licensing policy. These data are consistent with a conclusion that the on-road driving test for people aged 80 plus had little impact on mobility or safety at a population level, even though many individuals would have been affected. The lack of safety effects is consistent with findings comparing jurisdictions with contrasting older driver licence renewal procedures [12,13].

This result can be seen as an apparent conflict with previous research on older drivers in New Zealand and the association between their performance in the on-road test and subsequent crash involvement rates [1]. In that study, each driving test failure was found to be associated with a 33% increase in the odds of crash involvement in the two years following the driving test (95% CI 14% to 55%), controlling for age, gender, minor traffic violations, and whether the older driver lived with another licensed driver or not. This demonstrated that the on-road test did identify aspects of driving behaviour that led to higher crash involvement risk. An explanation why the current study did not find changes in crash rates is that only a small group of drivers would have been directly affected by the regulation change. As noted above, about 5% of drivers aged 80 attempted licensing annually but failed the on-road driving test when it was a requirement of licensing [1]. It can be assumed that another group of unknown size did not even attempt licensure, deterred by the stress of the on-road test. As cognitively intact older drivers are known to regulate their driving when they perceive that their capability is diminishing [22], the group of drivers most affected by the regulation change were likely to drive less than average, so changes in mobility and safety may still have occurred because of the licensing policy change, but been too small to be detected by the population-level analysis undertaken.

The current study has strengths and limitations. Strengths include the wide range of data sources that were available to be analysed. Weaknesses included the relatively small sample sizes for the group affected by the policy change and associated lack of power to detect real changes (if any). This was compounded in some cases by reliance on published data that combined the group of interest (those aged 80 plus) with drivers aged 75–79. Additionally, the travel survey estimates using self-reported travel are likely to underestimate actual travel. In a study of eight countries, comparisons of reported physical activity with pedometer readings showed only fair agreement between these measures [23]. Similarly, older people have been found to underreport driving activity relative to GPS measures of actual driving [24]. However, comparisons made over time between age groups, such as analysed in this study, should be relatively robust to modest levels of reporting bias.

## Conclusions

Despite the limitations outlined above, it can be concluded that the evidence available is inconsistent with major changes in safety or mobility at the time of the removal of the older driver on-road test as a general condition of licensing. Along with other relevant studies comparing policies across different jurisdictions, this study does not suggest any safety benefits from the generalised use of on-road testing as an assessment mechanism for all older drivers.

## Competing interests

The authors declare that they have no competing financial or other interests.

## Authors’ contributions

MK conceived the study, collated the data sources, undertook the analysis, and was the primary author of the manuscript. EW contributed to drafting the paper. Both authors read and approved the final manuscript.

## Pre-publication history

The pre-publication history for this paper can be accessed here:

http://www.biomedcentral.com/1471-2458/14/165/prepub
